# Comparison of lactate values obtained from different sites and their clinical significance in patients with severe sepsis

**DOI:** 10.1590/S1516-31802011000100003

**Published:** 2011-01-06

**Authors:** Ana Paula Metran Nascente, Murillo Assunção, Carla Janaina Guedes, Flávio Geraldo Rezende Freitas, Bruno Franco Mazza, Miriam Jackiu, Flávia Ribeiro Machado

**Affiliations:** I MD. Attending physician in the Intensive Care Unit, Discipline of Anesthesiology, Pain and Intensive Care, Universidade Federal de São Paulo — Escola Paulista de Medicina (Unifesp-EPM), São Paulo, Brazil.; II MD, MSc. Coordinator of the Intensive Care Unit, Discipline of Anesthesiology, Pain and Intensive Care, Universidade Federal de São Paulo — Escola Paulista de Medicina (Unifesp-EPM), São Paulo, Brazil.; III MD. Coordinator of the Intensive Care Unit, Discipline of Anesthesiology, Pain and Intensive Care, Universidade Federal de São Paulo — Escola Paulista de Medicina (Unifesp-EPM), São Paulo, Brazil.; IV MD, PhD. Adjunct professor, Discipline of Anesthesiology, Pain and Intensive Care, Universidade Federal de São Paulo — Escola Paulista de Medicina (Unifesp-EPM), São Paulo, Brazil.

**Keywords:** Lactic acid, Perfusion, Sepsis, Shock, septic, Hemodynamics, Ácido láctico, Perfusão, Sepse, Choque séptico, Hemodinâmica

## Abstract

**CONTEXT AND OBJECTIVE:**

The ideal site for lactate collection has not been clearly established. This study aimed to evaluate associations between lactate levels in arterial blood (Lart), peripheral venous blood (Lper) and central venous blood (Lcen) in patients with severe sepsis or septic shock.

**DESIGN AND SETTING:**

Cross-sectional analytical study in an tertiary university hospital.

**METHOD:**

Samples from patients with a central venous catheter and from healthy volunteers (control group) were collected. Blood was drawn simultaneously for measurements of Lart, Lper and Lcen, and the first sample was collected less than 24 hours after the onset of organ dysfunction. The results were analyzed using Pearson correlation, Bland-Altman and McNemar tests.

**RESULTS:**

A total of 238 samples were collected from 32 patients. The correlation results were r = 0.79 (P < 0.0001) for Lart/Lper and r = 0.84 (P < 0.0001) for Lart/Lcen. Bland-Altman showed large limits of agreement: -3.2 ± 4.9 (-12.8 to 6.4) and -0.8 ± 5.9 (-12.5 to 10.8), for Lper and Lcen respectively. In the control group, there was greater correlation (r = 0.9009, P = 0.0004) and agreement: -0.7 ± 1.2 (-3.1 to 1.7). Regarding clinical intervention, there was good agreement between Lart/Lcen (96.3%; three disagreements), with worst results for Lart/Lper (87.0%) with 10 cases of disagreement (P = 0.04). In eight patients (80.0%) Lper was higher than Lart.

**CONCLUSION:**

Lcen, and not Lper, can replace Lart with good correlation and clinical agreement. Lper tends to overestimate Lart, thus leading to unnecessary therapeutic interventions.

## RESUMO

**CONTEXTO E OBJETIVO:**

O sítio ideal de coleta do lactato não está claramente estabelecido. Este estudo objetivou avaliar a associação dos valores de lactato do sangue arterial (Lart), venoso periférico (Lper) e venoso central (Lcen) em pacientes com sepse grave ou choque séptico.

**TIPO DE ESTUDO E LOCAL:**

Estudo transversal analítico em hospital universitário terciário.

**MÉTODOS:**

Amostras de pacientes com cateter venoso central e voluntários sadios (grupo controle) foram coletadas. O sangue foi obtido de forma simultânea para medida do Lart, Lper and Lcen, sendo a primeira amostra coletada no máximo 24 horas após o início da disfunção orgânica. Resultados foram analisados usando a correlação de Pearson, os testes de Bland-Altman e McNemar.

**RESULTADOS:**

Um total de 238 amostras foi coletado em 32 pacientes. Resultados da correlação: r = 0.79 (P < 0,0001) para Lart/Lper e r = 0.84 (P < 0,0001) para Lart/Lcen. Bland-Altman mostrou largos limites de concordância, -3.2 ± 4.9 (-12.8 to 6.4) and -0.8 ± 5.9 (-12.5 to 10.8), para Lper e Lcen, respectivamente. No grupo controle, houve uma maior correlação (r = 0.9009, P = 0,0004) e concordância: -0.7 ± 1.2 (-3.1 to 1.7). No que se refere à intervenção clínica, houve boa concordância Lart/Lcen (96.3%, três não concordantes), com resultados piores encontrados para Lart/Lper (87.0%) com 10 casos não concordantes (P = 0,04). Em oito pacientes (80.0%) Lper foi mais elevado que Lart.

**CONCLUSÃO:**

Lcen, e não Lper, pode substituir Lart com boa correlação e concordância clínica. Lper tende a superestimar Lart, assim levando a intervenções terapêuticas desnecessárias.

## INTRODUCTION

Sepsis is a disease characterized by hypercatabolism with increased demand for oxygen due to elevated consumption in tissue. When an imbalance between the supply (DO2) and consumption (VO2) of oxygen is present, tissue hypoperfusion and hypoxia lead to anaerobic metabolism with final production of lactate. Early detection of this status is crucial, since it is well known that early therapy with optimization of blood volume, hemoglobin levels and/or use of inotropic agents favors the patient's prognosis.^1^

Despite questioning relating to the mechanisms of hyperlactatemia,^2-5^ this is a well-recognized instrument for diagnosing hypoperfusion and occult tissue hypoxia, and it is also used as a prognostic index among septic patients.^6,7^ However, although hyperlactatemia is generally measured in the arterial blood, the ideal collection site has not been clearly established.

Taking into account the lactate synthesis and clearance mechanisms, the collection site in critical patients may, theoretically, inter-fere with the interpretation of results and lead to inadequate management. Lactate measured in samples collected from arterial blood (Lart) would best represent the overall perfusion since such samples contain blood coming from the pulmonary veins, superior vena cava and infe-rior vena cava. Peripheral lactate (Lper) may preferentially reflect the perfusion and metabolism of the compartment from which the blood was drawn (i.e. the local perfusion) but not the overall perfusion. On the other hand, although lactate measured in blood drawn from the superior vena cava (Lcen) is regarded as an overall measurement, it may not appropriately represent the perfusion in the lungs and gastrointestinal tract.^8,9^

Previous studies were carried out in order to detect differences between lactate levels in samples collected from different sites. Good correlations were found between arterial and central venous or mixed blood, along with narrow limits of agreement.^10-12^ Weil et al. found a good linear correlation between the Lart and Lcen levels and between Lart and mixed venous lactate.^10^ More recently, Middleton et al also reported good agreement between the Lart and Lcen values in critically ill patients.^12^ However, Evron et al. evaluated a larger group of patients undergoing major surgery and found that the Lper levels were higher than the Lcen and Lart levels. Likewise, the Lcen levels were higher than the Lart levels. However, these authors did not carry out agreement or correlation tests.^13^ Other authors have shown narrow limits of agreement between Lart and Lper, despite weak correlation. Once again, the samples collected from peripheral venous sites generally presented higher values.^14^ Adams and Hazard showed good correlation between Lper and Lart, although the degree of agreement was not analyzed.^14^ However, some of these studies are old, with small samples,^10,14,15^ and none of them sought to evaluate the impact on the clinical management or specifically analyzed septic patients.^10-15^ Comparison with samples from healthy volunteers as controls was also not carried out in the previously mentioned studies.

## OBJECTIVE

The purpose of this study was to evaluate the association between lactate values obtained from different collection sites in a specific population of patients with severe sepsis and septic shock, with emphasis on the impact on medical management.

## METHODS

### Type of study

This was a cross-sectional analytical study with a control group that was carried out in the intensive care unit of the Discipline of Anesthesiology, Pain and Intensive Care, Hospital São Paulo, Universidade Federal de São Paulo — Escola Paulista de Medicina (Unifesp-EPM), which is a tertiary-level public institution. The research project had previously been analyzed and approved by the institution's Ethics Committee. A free and informed consent statement was obtained, signed by all participating patients or their legal representatives.

### Sample

Patients could be included if they were in the intensive care unit with a diagnosis of severe sepsis and septic shock, in accordance with the definitions of the 1992 ACCP/SCCM consensus.^16^ Organ dysfunction needed to be acutely associated with the sepsis episode. The criteria are shown in **Chart 1**.

Chart 1. **Criteria for defining organ dysfunction**

- Arterial hypotension (SBP < 90 mmHg, MAP < 70 or SBP decrease > 40 mmHg).
- Arterial hypoxemia (PaO_2_/FiO_2_ < 300).
- Acute oliguria (urine output < 0.5 ml/kg/hr for at least 2 hours) or creatinine > 2.0 mg/dl
- Altered mental status (Glasgow coma scale < 13).
- Thrombocytopenia (platelet count < 100,000 cells/mm^3^).
- Hyperbilirubinemia (plasma total bilirubin > 2.0 mg/dl)
- Unexpected metabolic acidosis (pH ≤ 7.30 or base excess ≥ 5.0 mEq/l)
- Coagulation abnormalities (INR > 1.5 or aPTT > 60 sec)

SBP = systolic blood pressure; MAP = mean arterial pressure; INR = international normatization ratio; aPTT = partial activated thromboplastin time. PaO_2_/FiO_2_ = arterial oxygen partial pressure/fraction of inspired oxygen ratio.

The inclusion criteria were: onset of organ dysfunction less than 24 hours after the first sample collection; indication for or presence of a central venous catheter; signing of an informed consent statement by the patient or his/her legal representative; and age over 18 years. The exclusion criteria were the presence of chronic or acute hepatic failure and the need for dialysis. The control group was composed of healthy volunteers.

### Procedures

Blood was drawn simultaneously for measurements of Lart, Lper and Lcen, every 12 hours, with a maximum of three collections per patient. For the Lart collection, 1 ml of blood was obtained through arterial puncture or from an arterial line. A tourniquet was applied to the upper limb for up to two minutes and 1 ml of peripheral venous blood was obtained for Lper determination. Lcen was obtained through collecting 1 ml of blood from a central venous catheter placed in the sub-clavian or internal jugular vein, after 5 ml of blood had been aspirated and discarded. The position of the catheter was checked by means of chest x-ray.

The samples collected from the different sites were sent for measurement of lactate levels, and also arterial and central venous gas analysis, in order to ensure that the collection site was arterial and to allow sub-group analysis based on central venous saturation, respectively. All measurements were performed no more than 30 minutes after sample collection. Lactate levels were determined by enzyme colorimetric assay.

To analyze the concordance of medical management a board-certified intensivist who was blinded to the site of blood collection, defined the management approach according to each sample. In this, the unit protocol based on the resuscitation bundle of the Surviving Sepsis campaign was followed.^17^ Each patient's hemodynamic data, i.e. hemoglobin levels, blood pressure, heart rate, central venous pressure, central venous oxygen saturation, urinary output and doses of vasoactive drugs, were taken into account. The blinded intensivist performed two repeated analyses and an intra-observer agreement rate was determined.

The correlation and agreement between the lactate values were analyzed in subgroups of patients according to the presence of respiratory dysfunction, septic shock and central venous oxygen saturation.

### Statistical methods

The sample size calculation was carried out using the Stplan software (version 4.3), based on the possible correlation between Lart and Lper, in a two-sided manner, with a significance level of 0.05 and statistical power of 80%. The population correlation coefficient used was 0.7, and 0.5 was taken to be the null hypothesis correlation. The calculated number of samples was 81 pairs.

The normality of the lactate values was assessed using the Shapiro-Wilk test, and this showed that the distribution was non-normal. Consequently, the results were expressed as medians, together with the inter-quartile range (25% to 75%).

Pearson's correlations were calculated using Lart as a reference for comparisons with both Lcen and Lper. The results were expressed through the correlation coefficient (R) and the descriptive level (p) to test its significance. Additionally, the Bland-Altman test was used to determine the bias and the limits of agreement between Lcen and Lper, compared with the reference value (Lart). The results from the Bland-Altman test were expressed as bias ± standard deviation (95% confidence interval), and this confidence interval represented the limits of agreement.

The concordance of clinical management was expressed as percentages. Additionally, a comparison of the concordance of Lper with Lart versus that of Lcen with Lart was performed through the McNemar test. In all the tests, the results were considered statistically significant if the P descriptive level was ≤ 0.05.

## RESULTS

A total of 238 lactate measurements were made on 32 non-consecutive patients, with a mean age of 59.9 ± 17.9, of whom 13 were women. The demographic and diagnostic characteristics are shown in **Table 1**. All patients included in the study had a diagnosis of severe sepsis or septic shock not more than 24 hours prior to enrollment. The mean values of the acute physiological and chronic health evaluation (APACHE) II and sequential organ failure assessment (SOFA) scores were 15.8 ± 6.8 and 6.6 ± 3.4, respectively. The control group was composed of 10 healthy volunteers (mean age 30.3 ± 3.9; three males and seven females).

**Table 1.** Patients' baseline characteristics

Characteristic	Results
Age (years)*	59.9 ± 17.9
Gender	
male	19 (59.4)
female	13 (40.6)
Race	
white	29 (90.6)
black	3 (9.4)
Category	
Severe sepsis	17 (53.1)
Septic shock	15 (46.9)
APACHE II score*	15.8 ± 6.8
SOFA score*	6.6 ± 3.4
Admission type	
clinical	8 (25.0)
elective surgery	5 (15.6)
emergency surgery	19 (59.4)
Comorbidities	
cardiovascular	15 (46.9)
respiratory	8 (25.0)

APACHE = Acute Physiological and Chronic Health Evaluation; SOFA = Sequential Organ Failure Assessment.

Results are expressed as number (percentage) except for * expressed as mean ± standard deviation.

From 17 patients, three sets of measurements were obtained, while from the other 15 patients, only two sets were collected. Each set was composed of three samples, which generated 80 paired Lart/Lcen samples and 77 paired Lper/Lart samples. In relation to four patients, Lper sampling was not possible for technical reasons and in one patient, the Lcen sample was lost. The median lactate values were 14.0 (11.0-21.0) mg/dl for Lart, 16.2 (12.5-22.3)mg/dl for Lper and 14.0 (11.0-19.9) mg/dl for Lcen. In the control group, the median Lart was 12.5 mg/dl (range: 10.0-15.0) and the median Lper was 12.0 mg/dl (range 11.0-16.0).

**Figure 1:**
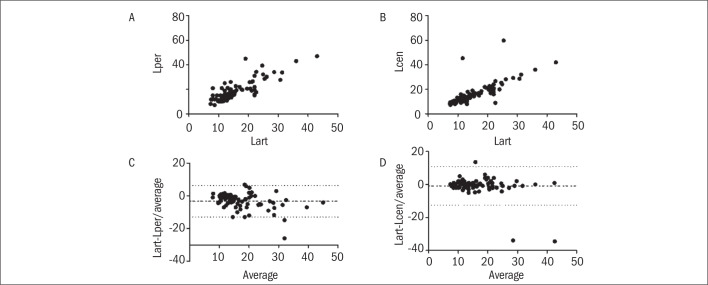
Results from the study group.

**Figure 2.**
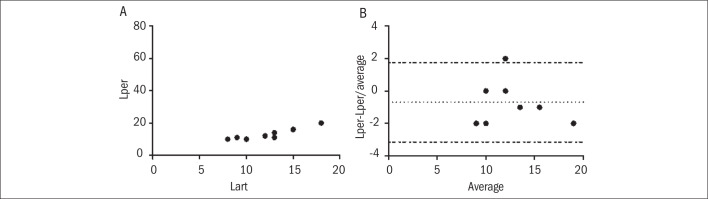
Results from the control group.

In the study group, a moderate linear correlation was found between the levels of arterial and peripheral venous lactate, with r = 0.79 and P < 0.0001 (**Figure 1A**). This correlation was stronger between Lart and Lcen (r = 0.84, P < 0.0001) (**Figure 1B**). However, the Bland-Altman analysis showed very wide limits of agreement. When the Lart and Lper results were compared, the mean difference was -3.2 ± 4.9, with limits of agreement between -12.8 and 6.4 (**Figure 1C**). Likewise, when the Lart and Lcen values were compared, the mean difference was -0.8 ± 5.9 with limits of agreement between -12.5 and 10.8 (**Figure 1D**). The control group showed a better linear correlation between the Lart levels, with r = 0.9009 and P = 0.0004 (**Figure 2A**). In the Bland-Altman test, the mean difference was -0.7 ± 1.2 (-3.1 to 1.7), which showed that there was good agreement between the methods (**Figure 2B**).

Linear correlations and Bland-Altman tests were performed in the subgroups of patients according to the presence of respiratory dysfunction, septic shock and central venous blood saturation. In cases of septic shock, there seemed to be more variability in Lper than in Lart (-3.3 ± 4.5 (-12.1 to 5.4) and -2.6 ± 3.9 (-10.2 to 5.1) for the presence or absence of shock, respectively), but not for Lcen compared with Lart. Similarly, patients with respiratory dysfunction seemed to have less agreement between Lart and Lper (-3.6 ± 4.4 (-12.3 to 5.0) and -1.6 ± 3.4 (-8.4 to 5.1), with and without dysfunction, respectively) but not between Lart and Lcen. However, this profile was not maintained for SvO_2_, since the agreement in patients with saturation lower than 70% did not change either for Lper or for Lcen (**Table 2**).

**Table 2.** Comparative analysis between lactate values from different sites, in subgroups

Variables	Present	Absent
Septic shock		
Lper	-3.2 ± 4.5 (-12.1 to 5.4)	-2.6 ± 3.9 (-10.2 to 5.1)
Lcen	0.4 ± 2.1 (-3.8 to 4.5)	-0.4 ± 23.0 (-6.2 to 5.5)
Respiratory dysfunction		
Lper	-3.6 ± 4.4 (-12.3 to 5.0)	-1.6 ± 3.4 (-8.4 to 5.1)
Lcen	-0.4 ± 2.0 (-4.4 to 3.5)	0.8 ± 3.3 (-5.7 to 7.3)
SvO_2_ < 70%		
Lper	-1.7 ± 4.2 (-10.0 to 6.5)	-2.9 ± 4.0 (-10.7 to 4.8)
Lcen	0.5 ± 3.9 (-7.1 to 8.1)	-0.2 ± 2.2 (-4.6 to 4.2)
Overall		
Lper	-3.2 ± 4.9 (-12.8 to 6.4)	
Lcen	-0.8 ± 5.9 (-12.5 to 10.8)	

Lper = peripheral venous lactate; Lcen = central venous lactate; SvO_2_ = venous oxygen saturation. Bland-Altman results are expressed as bias ± standard deviation (limits of concordance), all of them compared with arterial lactate.

There was good agreement regarding the medical management for Lart and Lcen (96.3%; three discordant results). For Lper, this agreement was lower (87.0%), given that in 10 cases, the medical management generated by Lper was different from the management generated by the Lart result. Of these, only two cases also had a discordant result regarding Lcen measurement. Therefore, in eight cases there was concordance of Lart/Lcen and discordance of Lart and the peripheral measurement. The McNemar test, applied to comparison of concordances of central venous and peripheral blood (with arterial blood as the reference) showed a significant result, with P = 0.04 (**Table 3**), suggesting that Lcen is more appropriate than Lper for clinical management. In a detailed analysis on ten cases with no clinical agreement between Lper and Lart, it was observed that eight patients (80.0%) presented Lper results higher than those of Lart. The reproducibility of this analysis was good, with intra-observer variability of 3.3%.

**Table 3.** Comparison between central venous and peripheral lactate regarding to their clinical concordance with arterial lactate

Lper/Lart concordance	Lcen/Lart concordance	
	Yes	No
Yes	66	1
No	8	2

Lper = peripheral venous lactate; Lcen = central venous lactate; Lart = arterial lactate. Yes or no refers to concordance with clinical management based on arterial lactate. Results are expressed as number of paired samples. McNemar – P = 0.04.

## DISCUSSION

This study showed that lactate sampling from central venous blood presented good correlation (r = 0.84, P < 0.0001) and reasonable agreement with arterial lactate levels. Additionally, there was good concordance between arterial and central venous-based medical management. However, in terms of Lper, a moderate correlation was shown (r = 0.79, P < 0.0001), with broad limits of agreement in the Bland-Altman test and lower clinical concordance with Lart-based management.

These results are similar to those found by other authors, which showed high correlation coefficients between the Lart and Lcen measurements^10^ or mixed central venous measurement.^10-11^ However, the limits of agreement found here in the Bland-Altman tests were higher than those reported by Murdoch et al.^11^ Regarding Lper, our results showed much higher bias and limits of agreement than in a previous study.^14^ It is possible that the samples in previous studies, which were smaller than the current one, may have been insufficient to show the real difference between the Lart and Lcen/Lper measurements, with a trend towards minimizing it. In the current study, with a larger case load, such values were less concordant, particularly the Lper results.

It would be expected that the lactate levels in arterial blood would be higher than in central venous blood, since arterial blood samples are more representative of overall blood lactate. Venous drainage from the splanchnic area, coronary sinus and lungs is not represented in the central venous blood. Previous studies on patients with acute pulmonary lesions and on septic patients showed that there is regional pulmonary production of lactate, and that the levels increase according to the severity of the pulmonary lesions.^3,8^ However, the present study showed similar results for Lart and Lcen. Even in the subgroup with respiratory dysfunction, no significant difference in agreement could be found.

The same cannot be said about lactate from peripheral puncture. The reason why Lper does not reflect systemic perfusion as well as Lcen does is possibly related to interference from regional perfusion of the upper limb where the collection took place. Attention was paid to timely collection of this material, and a tourniquet was used in the upper limb for up to two minutes. As expected, the median value of Lper (16.2 mg/dl) was higher than the median lactate value collected from the other sites (Lcen and Lart, 14.0 and 14.0, respectively), since the circulation in situations of septic status is deviated towards the critical organs. Moreover, larger limits of agreement between Lart and Lper were seen in the subgroup of patients with septic shock, thus suggesting that peripheral vasoconstriction due to vasopressors can further impair the relationship between Lper and the overall perfusion. It is worth noting that even in this scenario, Lcen performed well. In the control group, which was composed of healthy volunteers, the correlation between the Lart and Lper values was higher, possibly because of the normal conditions of tissue perfusion and homogeneity present in healthy individuals. Unfortunately, it was not possible to evaluate the relationship with Lcen among the healthy controls because of the absence of a central venous catheter.

This limitation of Lper was confirmed by the clinical agreement analysis, since Lcen performed better than Lper (taking Lart-based clinical management as the gold standard). Moreover, among the 10 patients who showed discordant medical management between Lart and Lper, eight had higher results for Lper. Therefore, because Lper overestimates Lart, this may lead to therapeutic interventions in patients who do not need treatment.

Given the results presented by the current study, we propose that Lart data should be collected for perfusion assessment among septic patients. Lart can be replaced by Lcen if a central line, rather than an arterial line, is available. Despite the easier sampling from peripheral sites in the emergency room, such sampling may not be an appropriate option. In this setting, the initial lactate measurement will determine whether or not an intervention aimed at hemodynamic optimization is needed.^1,18^ As demonstrated here, Lper can overestimate the Lart results. Interventions such as volume replacement or dobutamine administration are not exempt from side effects. Recent studies have shown that a positive water balance is related to intra-abdominal hypertension and acute respiratory distress syndrome. It has also been postulated that the interstitial edema produced has a negative influence on the oxygen diffusion to the tissues, because of the increased distance between the capillaries and the tissue.^19,20^

This study presents certain strengths. First, it was carried out in a prospective manner. Second, compared with previous studies, it included a more homogeneous population and analyzed a larger case load only consisting of septic patients. Third, the patients were at a very early phase of sepsis, i.e. at a stage at which lactate levels are crucial for management and differences between Lart and Lper are potentially greater. Fourth, it evaluated both statistical characteristics (with subgroup analysis) and clinical characteristics, in a blinded manner. There are no previous studies analyzing clinical importance in terms of lactate values collected from different sites, and agreement regarding medical management is probably more relevant than coefficients of correlation or limits of agreement, at least from the point of view of conducting a clinical case. Fifth, it has a control group, thus enabling agreement analysis without the presence of hypoperfusion.

This study also has certain limitations. First, our control group was small, possibly compromising the power of our comparison analysis. It also had a lower mean age than the study group. However, we do not think that age can modify lactate levels or, even less, the relationship between lactate samples collected from different sites. Second, there were three samples with very disparate values from their paired Lart samples (two Lcen and one Lper sample). We cannot clearly explain these findings. It is possible that these levels represent the true Lcen and Lper values at the moment of sampling, thus reinforcing the possible disagreement with arterial blood sampling. However, we cannot rule out the possibility of laboratory errors, although care was taken to avoid delays in the analyses as well as the possibility of misplacing the tests. In the case of Lper, it is possible that, even with all the precautions taken, the duration of tourniquet use could have been excessive.

## CONCLUSION

In septic patients, Lcen may replace Lart collection, with a good correlation between them and, especially, good concordance regarding medical management. The same cannot be said about Lper because, despite a reasonable correlation with Lart, it tends to overestimate Lart, which may lead to unnecessary therapeutic interventions for such patients. The difference found between Lart and Lper may be caused by metabolic abnormalities or the perfusion that is present in the early stages of sepsis.
